# Melatonin and TGF-β-Mediated Release of Extracellular Vesicles

**DOI:** 10.3390/metabo13040575

**Published:** 2023-04-18

**Authors:** Klaudia Piekarska, Klaudia Bonowicz, Alina Grzanka, Łukasz M. Jaworski, Russel J. Reiter, Andrzej T. Slominski, Kerstin Steinbrink, Konrad Kleszczyński, Maciej Gagat

**Affiliations:** 1 Department of Histology and Embryology, Collegium Medicum in Bydgoszcz, Nicolaus Copernicus University in Torun, 85-092 Bydgoszcz, Poland; klaudia.mikolajczyk@cm.umk.pl (K.P.); klaudia.bonowicz@cm.umk.pl (K.B.); agrzanka@cm.umk.pl (A.G.); lukaszmjaworski@gmail.com (Ł.M.J.); 2 Department of Cell Systems and Anatomy, UT Health, Long School of Medicine, San Antonio, TX 78229, USA; reiter@uthscsa.edu; 3 Department of Dermatology, Comprehensive Cancer Center, University of Alabama at Birmingham, Birmingham, AL 35294, USA; aslominski@uabmc.edu; 4 Pathology and Laboratory Medicine Service, VA Medical Center, Birmingham, AL 35294, USA; 5 Department of Dermatology, University of Münster, Von-Esmarch-Str. 58, 48149 Münster, Germany; kerstin.steinbrink@ukmuenster.de

**Keywords:** melatonin, transforming growth factor β, extracellular vesicles, cell-to-cell communication

## Abstract

The immune system, unlike other systems, must be flexible and able to “adapt” to fully cope with lurking dangers. The transition from intracorporeal balance to homeostasis disruption is associated with activation of inflammatory signaling pathways, which causes modulation of the immunology response. Chemotactic cytokines, signaling molecules, and extracellular vesicles act as critical mediators of inflammation and participate in intercellular communication, conditioning the immune system’s proper response. Among the well-known cytokines allowing for the development and proper functioning of the immune system by mediating cell survival and cell-death-inducing signaling, the tumor necrosis factor α (TNF-α) and transforming growth factor β (TGF-β) are noteworthy. The high bloodstream concentration of those pleiotropic cytokines can be characterized by anti- and pro-inflammatory activity, considering the powerful anti-inflammatory and anti-oxidative stress capabilities of TGF-β known from the literature. Together with the chemokines, the immune system response is also influenced by biologically active chemicals, such as melatonin. The enhanced cellular communication shows the relationship between the TGF-β signaling pathway and the extracellular vesicles (EVs) secreted under the influence of melatonin. This review outlines the findings on melatonin activity on TGF-β-dependent inflammatory response regulation in cell-to-cell communication leading to secretion of the different EV populations.

## 1. Introduction

The proper functioning of the cells that build the vessel walls is the basic condition for maintaining homeostasis in the body [1,2,3]. In the heart, arteries, capillaries, and veins the multi-functional nature of the ECs relies on providing an anti-inflammatory and anti-coagulatory surface in the physiological state for the remaining cells [4,5]. On the other hand, the vessel wall layer controls the adhesion and migration of inflammatory cells under imbalanced conditions. Any disturbance that causes the disruption of intercellular connections of ECs and vessel unsealing may lead to leakage of immune cells from the lumen to adjacent tissues, and initiation of inflammation [6,7,8]. Typically, this process is part of the innate immunity and physiological response to injury; however, if prolonged, it constitutes a major factor in the development and complications of atherosclerotic cardiovascular diseases. For this reason, anti-inflammatory therapies involving the stabilization of chemotactic cytokines are the current trend in cardiovascular medicine [9,10,11].

Chemotactic cytokines, also known as chemokines, are a group of proteins that stimulate the movement of leukocytes and control their migration from the blood to tissues [12]. This property determines their undeniable role in the formation of an inflammatory focus. The altered concentration of chemokines in individual disease states may be the target of research—as potential diagnostic or prognostic markers, as well as a promising target for therapeutic interventions [13,14].

At initiation sites of inflammation, chemokines direct the progression of the immune response based on the leukocyte migration across the endothelium [15,16]. The inflammatory reaction is a multi-stage process controlled by the interaction of adhesion molecules located on the luminal surface of endothelial cells with surface leukocyte receptors [17,18]. Chemokines mobilize the immune system cells to concentrate at the focus of inflammation and maintain homeostasis of the body. This process is referred to as extravasation, which involves a cascade of reactions where the first step is the contact of leukocytes with the EC layer, which is called leukocyte rolling [19]. During slow rolling, leukocytes can interact with the chemoattractant present on the surface of the endothelium, which binds to specific transmembrane receptors linked to intracellular Gi proteins [20]. Signals transmitted by this class of receptors increase the affinity of integrins, which ensures the stable adhesion of leukocytes to endothelial cells. Then, integrins can bind to adhesive proteins, e.g., intercellular adhesion molecule 1 (ICAM-1), and vascular cell adhesion molecule 1 (VCAM-1) [21,22]. The next step is cytokine-dependent activation and selectin-dependent tight adhesion, which consequently allows cells to pass through the endothelial layer to the surrounding tissues by diapedesis [23]. The first stages depend mainly on selectins, including *E*- and *P*-selectins, which alternately bind briefly and release from bonds to carbohydrate groups, slowing down the movement of leukocytes in the vessel [24]. Their expression is regulated by cytokines, while their ligands are expressed on specific leukocyte subpopulations [25,26,27]. The expression of selectins and selectin ligands is limited to the microvilli present on the surface of leukocytes, allowing for effective interaction with vascular ECs. The chemokine activity is therefore essential for the initiation and course of a proper immune system response and regaining internal balance [12,28]. Chemokines play an extremely important role in the development of cardiovascular diseases, i.e., the progression of atherosclerotic plaque [29,30]. The initial stages of atherogenesis are associated with the exposure of the CXC chemokine ligand (CXCL) by ECs, which are regulated by lysophosphatidic acid, a component of low-density lipoproteins (LDL) [31]. For example, the chemokine CXCL1 may recruit leukocytes to infiltrate the vascular wall and influence the progression of atherosclerosis in response to stimulation by phosphatidic acid (PA) [32,33]. On the other hand, CCL17 inhibits the influence of regulatory T cells in the promotion of atherosclerotic lesions [34,35]. The expression of the CXCL12 chemokine in endothelial cells, which stabilizes atherosclerotic plaques, can be induced by microribonucleic acid (miR)-126 [36,37]. Another chemokine receptor, CX3CR1, is responsible for sending strong signals that prolong the survival of monocytes and macrophages, which protects them from apoptosis. In contrast, CXCL5 reduces the formation of foam cells from macrophages [38,39,40,41]. Thus, the cytokine essential for many key cellular processes and for maintaining the homeostasis of every cell in the body, TGF-β, has been considered [42,43]. Despite many studies, its action is still difficult to characterize, due to its pleiotropic properties. It has been well-explained in cancer research and has been referred to as “the TGF-β paradox”. However, in cardiovascular medicine, the role of TGF-β is still ambiguous. On the one hand, its protective role is emphasized, and it is considered a major driver of vascular inflammation [44,45]. To date, misregulated TGF-β signaling in humans has been linked to the onset of vascular pathologies and cardiovascular diseases such as arteriovenous malformations (AVMs), aneurysms, atherosclerosis, cardiac fibrosis, vascular remodeling of the retina (retinopathy), and valvular heart disease [46,47].

Growing evidence suggests that melatonin synthesized in pinealocytes exerts protective effects against atherosclerosis-based vascular diseases, but these mechanisms are poorly understood [48,49]. Melatonin possesses anti-inflammatory capacities with benefits in protecting the structural and functional integrity of vascular endothelium against aging-, oxidative-stress-, lipopolysaccharide-, and ischemia-induced damage [50,51,52]. These profound effects are mainly exacerbated due to its antioxidant properties affecting the reduction of reactive oxygen species (ROS), which are the driving force of vascular pathology [53]. Despite some contradictions, most of the data claims that melatonin is a promising supplement that has no side effects [54]. Herein, we summarize the most established benefits of melatonin in the vascular system, focusing on the molecular mechanisms regulating the TGF-β signaling pathway. 

The TGF-β signaling mechanism can modify the extracellular vesicle (EV) secretion process, the evidence for which points to an important connection of EVs with inflammatory response biology [55]. EVs form a heterogeneous group of nanoparticles, providing an extremely important means of transmitting information between cells, without direct contact [56,57]. Recently, the intensity of research focused on EVs has significantly increased, paying particular attention to their activity in intercellular communication, for which bioactive molecules carried by vesicles between cells are responsible. The activity of the TGF-β signaling pathway in the course of the inflammatory response may regulate the secretion of membrane structures in order to modulate intercellular communication, allowing for the restoration of intracorporeal homeostasis [58,59]. Particularly interesting seems to be the currently little-known effect of melatonin on the cellular environment. The presence of this neuromolecule not only modulates the inflammatory response, but also affects the biogenesis, EV secretion amount, and composition of membrane vesicle cargo [60,61].

The purpose of this review is to provide a detailed description of the EV secretion dependent on the TGF-β signaling pathway mediated by melatonin. We focus on the molecular cargo and EVs’ association with disease and emerging strategies for their therapeutic exploitation.

## 2. Development and Progression of Vessel Wall Inflammation

The inflammation linked to the onset of atherosclerosis occurs between the layers of large and medium arteries, more specifically in the subendothelial space [62,63]. The endothelium is the innermost part of the blood vessels (arteries, veins, and capillaries) and consists of a single, semi-permeable layer of cells that is constantly regulated by local hemodynamic forces [64,65]. Areas of low endothelial shear stress (ESS) are the most common predictor of atherosclerotic plaque formation [66]. Low ESS, tangential stress due to the friction of the flowing blood on the endothelial surface, is also considered a focal pro-inflammatory stimulus, which contributes to endothelial dysfunction [67,68]. Another crucial factor important for maintaining endothelial homeostasis is the balance between vasodilation and vasoconstriction, mainly mediated by endothelium-derived nitric oxide (NO) bioavailability and other relaxing and contracting factors, such as angiotensin, endothelin-1 (ET-1) and oxidants [69,70]. NO production is highly dependent on the activity of the endothelial NO synthase (eNOS), also influenced by shear stress force on mechanoreceptors [71,72]. Therefore, oxidative stress-induced endothelial dysfunction, in terms of vasomotor disturbances, is the earliest event in atherogenesis, quickly followed by tissue repair mechanisms [73,74].

Disabled endothelium is leaky, adhesive, and unable to relax vascular smooth muscle cells. The disruption in the normal function of the endothelial cells is inseparably accompanied by a gradual infiltration of immune cells [75]. Simultaneously, released reactive oxygen species (ROS) induce the nuclear factor kappa-light-chain-enhancer of activated B cell (NF-κB) expression, which culminates in the increase in the expression of cytokines involved in further ROS production [76,77]. TNF-α is a key cytokine that inhibits endothelium-dependent nitric oxide (NO)-mediated vasorelaxation by activating sphingomyelinase, resulting in ^•^O_2_^−^ production in the ECs [78,79,80]. TNF-α is also a potent pro-inflammatory cytokine, which promotes inflammatory endothelial activation by upregulating the expression of VCAM-1 and ICAM-1, allowing lymphocyte and monocyte adhesion [81]. The monocytes then transmigrate to the subintimal space through the interaction of monocyte chemotactic protein-1 (MCP-1) with the CCR2 receptor, where they differentiate into macrophages [82,83]. A particularly important process for plaque formation is the internalization of cholesterol-rich oxidized lipoproteins by monocytes, giving them a foamy appearance and secreting local cytokines, as well as ROS [84,85,86]. Other types of immune cells, such as DCs, T cells, B cells, and neutrophils participate in intraplaque inflammation [87,88]. The perpetuation of pro-inflammatory and oxidative atherosclerotic stimuli results in the recruitment of more macrophages, mast cells, and activated T and B lymphocytes, which enhance vascular lesions, which in turn release cytokines (i.e., interleukin-1*β* (IL-1*β*), TNF-α), increase the leukocyte extravasation to the submembrane space and maintain chronic inflammation [89,90].

The artery wall structure also consists almost entirely of circumferentially oriented vascular smooth muscle cells (VSMC), surrounding the ECs and constituting the tunica media. The VSMC is involved in the crosstalk between immune cells and ECs during all stages of atherosclerosis [91,92]. ECs-derived relaxants such as NO lower the activity tone of VSMCs, leading to (flow-mediated) vessel dilation to counteract the initial increase in wall shear stress and contribute to pathological vascular remodeling [93,94,95].

## 3. The TGF-β Signaling Pathway in the Cardiovascular System

TGF-β is one of the crucial mediators in the pathophysiology of cardiovascular diseases such as atherosclerosis and abdominal aortic aneurysm (IAA) [96,97]. This highly complex polypeptide growth factor is also described as a multifunctional cytokine that elicits its effects in the vascular system via an influence on endothelial cells, smooth muscle cells, and regulation of extracellular matrix (ECM) deposition [98,99]. TGF-β family member proteins are involved in a large variety of cellular processes, including the induction of proliferation, apoptosis, migration, adhesion, ECM protein production, and cytoskeletal organization [100,101].

The perturbations in TGF-β signaling are linked to vascular-wall inflammation, thickening, and remodeling. The most abundant isoform of the family in the cardiovascular system is TGF-β1, present in ECs and VSMC populations, but also in the myofibroblasts, macrophages, and other hematopoietic cells. The outcome of cellular response to TGF-β depends on the signaling mechanisms regulated both extracellularly and intracellularly [102,103]. TGF-β is produced in an inactive form and stored in the ECM as part of a large latent complex (LLC) consisting of TGF-β, latency-associated peptide (LAP), and latent TGF-β binding protein (LTBP) [104,105,106,107]. The newly synthesized TGF-β binds to the pro-domain, called LAP propeptide via covalent and non-covalent linkage and forms a small latent complex (SLC) to keep the molecule in a biologically inactive state and to maintain a conformation suitable for dimerization [108,109]. LTBP connects with SLC through covalent bonding and targets and stabilizes LLC in ECM rich in fibrillin and fibronectin. The latent TGF-β activation process is dependent on the cell context and may result from a proteolytic cleavage within the LAP pro-domain, which can be stimulated by factors such as plasmin, cathepsin, matrix, and metalloproteinases and the subsequent release of the mature TGF-β and/or a conformational change in the LAP, allowing exposure of the TGF-β ligand [104,105,106,107,108,109]. Bioactive ligands and unmasked sites of TGF-β bind to a TGF-β type II receptor (TGF-βRII), also referred to as activin receptor-like kinases (ALKs) at the cell surface [110]. The activated TGF-βRII then recruits and activates the TGF-β type I receptor (TGF-βRI) by trans-phosphorylation [111]. TGF-β cellular responses are also regulated by TGF-βRIII (also termed β-glycan), which exhibits no enzymatic activity but is considered an important helper molecule that presents TGF-β to TGF-βRII and facilitates its binding [112,113]. In the canonical TGF-β signaling pathway, trans-phosphorylation of TGF-βRI induces phosphorylation of transcriptional effector proteins, receptor-activated small mothers against decapentaplegic (R-Smads) such as Smad2 and Smad3 [114,115,116].

In endothelial cells, low TGF-β concentrations in ECs can activate the Smad1/5/8-based pathway. The Smads classification also includes inhibitory Smads (I-Smads, Smad6/7). Upon phosphorylation, R-Smads associate with Smad4 (Co-Smad), enter the nucleus, and regulate the transcription of TGF-β responsive genes [117,118,119]. The Smad-independent pathways are also important for the response to TGF-β stimulation, and include the Ras homologous (Rho) protein family, Src homology 2 domain-containing transforming protein 1 (ShcA), Ras-related C3 botulinum toxin substrate (RAC), rat sarcoma virus (RAS) protein family, cell division control protein 42 homologs (CDC42), TNF-α receptor-associated factor 6 (TRAF6), phosphoinositide 3-kinase (PI3K), transforming growth factor beta-activated kinase 1 (TAK1), partitioning-defective protein 6 (PAR6), mitogen-activated protein kinase 1 (MAP3K1), protein phosphatase 2 (PP2A) and death-associated protein 6 (DAXX) [120,121,122,123,124,125].

## 4. Effect of Melatonin on the TGF-β Signaling

The elevated expression level of TGF-β1 mRNA is observed during the development and progression of a variety of vascular diseases, including coronary artery disease (CAD) [126,127]. The cellular response to the TGF-β1 stimulation also depends on its proper synthesis, secretion, and activation. TGF-β’s effect on blood vessel function is concentration-dependent. The pleiotropic actions of this cytokine on the ECs depend mainly on factors such as EC origin, serum composition, cell density, and the combination of TGF-β receptors expressed on the cell surface [128,129,130]. In vitro studies on HCAEC confirm that TGF-β1 overexpression significantly promotes apoptosis, while TGF-β1 siRNA significantly inhibits cell apoptosis [131]. Moreover, activation of endothelial TGF-β signaling is one of the primary drivers of atherosclerosis-associated vascular inflammation, contributing to endothelial activation and increased vascular permeability [132]. EC treatment with TGF-β induces the expression of a number of pro-inflammatory cytokines, chemokines and their receptors (including CCL2), leucocyte adhesion molecules including ICAM-1 and VCAM-1, and matrix metalloproteinases (MMP2) as well as fibronectin, a pro-inflammatory ECM component long linked to inflammation [133,134]. Based on these data, the inhibition of TGF-β1 expression may serve as a target for the treatment of different types of cardiovascular diseases [135,136].

Recent reports indicate that one of the potent inhibitors of TGF-β signaling is melatonin [137]. Melatonin, structurally determined as 5-methoxy *N*-acetyl tryptamine is an indoleamine nocturnally released by the pineal gland into the blood and cerebrospinal fluid [138]. The melatonin secretion mechanism has been fixed by the endogenous circadian rhythm generator, which is connected with the pineal gland in the suprachiasmatic nucleus (SCN) localized into the anterior hypothalamus [139,140]. Information about the lighting conditions of the environment reaches the pineal gland through a complex neural pathway starting in the retina and covers the following signaling itinerancy: retina → retino-hypothalamic tract → SCN → paraventricular nucleus → medial forebrain bundle → tectum diencephalon → intermediate-lateral nucleus of the spinal cord → superior cervical ganglion → postganglionic sympathetic fibers → pineal pinealocytes [141]. Tryptophan has been defined as the initial compound for the production of melatonin, which after hydroxylation and decarboxylation is converted into serotonin. The transformation of this chemical compound to melatonin is based on the activity of two crucial enzymes for the entire process. The first is *N*-acetyl-transferase (NAT), which catalyzes the serotonin *N*-acetylation, whereas hydroxy indole-*O*-methyltransferase (HIOMT) carries out o-methylation, leading directly to the formation of melatonin. The melatonin lipophilic structure determines its pleiotropic properties, which allow it to pass through all biological barriers in the body [142,143]. The hydrophobic structure also determines the possibility of interacting with several biochemical pathways and indirectly and directly affecting other tissues and cells. Due to its lipophilicity, melatonin concentrates in membranes including those of mitochondria, and in the cell’s nucleus [144]. The melatonin presence in the mitochondria is strongly associated with the participation of this hormone in the body’s immune reactions associated with disorders of homeostasis caused by oxidative stress [145]. This condition consists in disturbing the balance between the by-products of metabolic changes, i.e., reactive oxygen species (ROS), and the ability to remove them from the body [146]. Many publications report on the ability of melatonin to capture free radicals, thus protecting cells from their harmful effects. Melatonin enhances the activity of antioxidant enzymes, affecting the redox potential in various types of cells. Melatonin scavenges free radicals to form kynuramine compounds such as cyclic 3-hydroxymelatonin (C3-OHM) and *N*^1^-acetyl-5-methoxykynuramine (AMK), but also *N*^1^-acetyl-*N*^2^-formyl-5-methoxykynuramine (AFMK). As mentioned, melatonin can modulate the cell membranes’ redox potential by increasing antioxidant cellular defense, either enzymatic or non-enzymatic, but also by protecting key redox proteins such as thioredoxin 1 (Trx1) from the oxidative mechanism. It is a cascade reaction pathway, independent of the presence of receptors on the surface of other cells, which leads to reductions in the free radicals’ deleterious effects [147,148].

However, for the most part, immunoregulatory effects of melatonin are based on the interaction with membrane and nuclear receptors located in the central nervous system (CNS), eyesight organs, skin, digestive tract, liver, heart, arteries, kidneys, prostate gland, and uterus [149]. The mechanism of melatonin action by binding to membrane receptors is based on the reduction in cyclic adenosine monophosphate (cAMP) concentration, which affects the signaling pathways of a number of biological signals’ secondary transmitters. The significant engagement of cAMP, inositol trisphosphate (IP3), cyclic guanosine monophosphate (cGMP), diacylglycerol (DAG), or arachidonic acid leads to changing patterns of enzyme activities [150]. In addition, melatonin is involved in the transmission of information based on the release of calcium into the cytosol by stimulating the activity of phospholipase C, which catalyzes the hydrolysis process. As a result of this process, among others, IP3 is formed, passing to the plasma reticulum, where Ca^2+^ ions are stored, strongly stimulating the increased secretion of these ions [151]. Melatonin has an affinity for orphan nuclear receptors—retinoid orphan receptors/retinoid Z receptors. The activity of nuclear receptors particularly affects leukocytes, by inhibiting the action of 5-lipoxygenase, the enzyme responsible for cellular leukotriene biosynthesis from arachidonic acid, underlying inflammatory processes [152]. Another mechanism of melatonin action is based on binding to intracellular proteins, such as calmodulin, calreticulin, and tubulin, but its antioxidant properties also promote the creation of a melatonin-dependent antioxidant system [153,154].

Although melatonin plays a significant role in maintaining homeostasis and protecting tissue functional activity under exposure to unfavorable environmental conditions, a high concentration of this substance can cause a negative effect on physiological process courses [155]. An excessive melatonin amount can come from improper supplementation based on supraphysiological doses of melatonin or dysfunction of the organs responsible for the secretion of this hormone. This can cause circadian rhythm disorder, by imitating “artificial darkness” [156]. High concentrations of melatonin are associated with a high amount of its metabolites, which could have deleterious effects per se. Due to the knowledge of the pharmacodynamics of melatonin, the consequences of its high concentration may concern the signaling of the immune system, the central nervous system, platelet aggregation, and the cardiovascular system, as well as glucose metabolism, ending in carcinogenesis [157].

**Figure 1:**
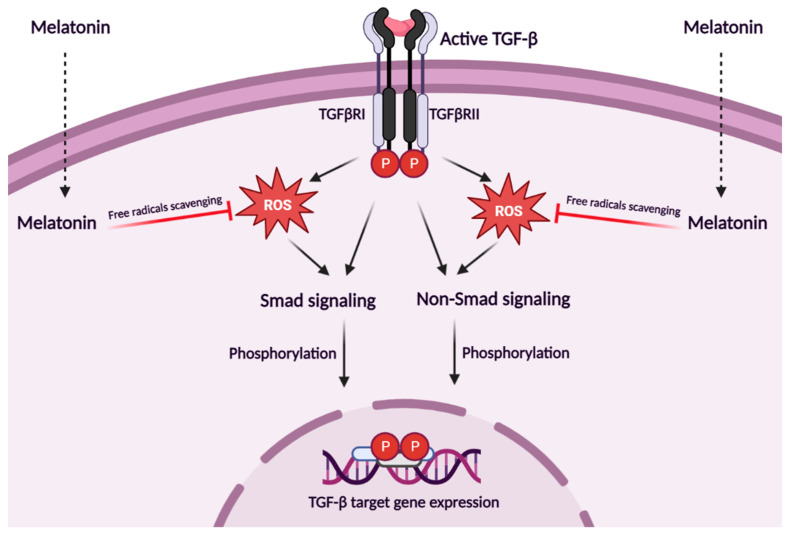
**Effect of melatonin on the TGF-β signaling.** TGF-β can signal via the canonical Smad proteins or in a Smads-independent manner. The specific course of the signaling pathway induced by the active TGF-β ligand depends on a series of phosphorylation of protein signal transducers. Intracellular levels of ROS are elevated after treatment with TGF-β1, while their presence ensures proper regulation of its signaling cascades. The hormone melatonin suppresses the TGF-β pathway due to its intracellular redox-status-altering properties, as evidenced by the effective reduction in ROS generation. Moreover, the inhibitory effect of melatonin is independent of its membrane receptor mechanisms. Indirectly, melatonin may interfere with many cellular processes coordinated by TGF-β-induced genes and intracellular ROS levels [158,159,160,161,162,163,164,165,166,167].

Pre-treatment with this indoleamine suppresses the increased intracellular level of ROS in TGF-β1-treated cells [158,159]. The antioxidant activity of melatonin can also attenuate epithelial-mesenchymal transition (EMT) stimulated by TGF-β1, by significant reversing changes in mRNA levels of *E*-cadherin, smooth muscle alpha-actin (α-SMA), vimentin, and fibronectin after TGF-β1 stimulation [160]. The TGF-β signaling pathway is mainly driven by a series of phosphorylation of Smad transducer proteins and their nuclear co-location to regulate the expression of target genes [161]. Melatonin prevents TGF-β1-induced cellular processes via the inhibition of Smad and non-Smad signaling cascades by inhibiting ROS-mediated mechanisms (Figure 1) [162]. Mechanistically, melatonin suppresses Smad2/3 phosphorylation and nuclear co-localization of their phosphorylated forms and Smad4 after TGF-β1 stimulation, in a dose-dependent manner [163,164]. The increasing phosphorylation of extracellular signal-regulated kinase 1/2 and p38 is attenuated by melatonin in a dose-dependent manner [165]. It is documented that the inhibitory action of melatonin does not require its membrane receptors [166]. The anti-inflammatory and anti-fibrotic actions of melatonin were also seen in the heart of melatonin-treated mice with diabetes mellitus, where it was found that melatonin significantly ameliorates cardiac dysfunction by inhibiting TGF-β1/Smad signaling and NOD-, LRR- and pyrin domain-containing protein 3 (NLRP3) inflammasome activation, as manifested by downregulating TGF-β1, *p*-Smad2, *p*-Smad3, NLRP3, ASC, cleaved caspase-1, mature IL-1*β*, and interleukin-18 (IL-18) [167].

## 5. Inflammatory EVs and Melatonin: Where Their Pathways Intersect

Different types of cells and tissues in the human body secrete distinct vesicles, which in turn allow for the transmission of intercellular signals through many pathways and, as a result, determine the maintenance of body homeostasis [168]. M137utual signal transduction via the membrane mediators is an important aspect of the body’s defense mechanisms because cells of the immune system are available to EVs for efficient and effective coordination of the immune response through the transport of biological molecules, primarily based on the proteins [169,170]. The course of immunological processes depends on the synchronization of a number of regulators of the immune system, both pro-inflammatory and acting as immune reaction brakes [171,172]. Among these biologically active pleiotropic compounds, significant importance has previously been ascribed to TGF-β, as well as the constitutive TNF-α molecule [173,174]. These substances mediate the secretion of membrane inflammatory mediators, affecting the frequency of their secretion, the number of secreted vesicles, and their molecular profile. Referring to the latest scientific reports, melatonin is also a factor that deserves special attention, as it can not only affect the mechanism of EV secretion but also modifies their cargo [175,176].

Melatonin is a widely acting anti-inflammatory molecule responsible for inhibiting chronic and acute inflammation, but it also removes ROS, which testifies to the antioxidant role of this compound [177,178]. The combination of properties characterized by EVs and melatonin-acting mechanisms seems to be a promising therapeutic strategy [179,180]. Accordingly, the melatonin-derived modification of the EVs cargo is considered a potential factor influencing damaged cells, noticeably modifying their molecular profile [181]. As a result, the presence of modified EVs in the environment of damaged cells can induce significant changes in the signaling mechanisms of these cells, often affecting their further fate [182].

### 5.1. Overview of Origin, Composition, and EVs Significance

EVs form a heterogeneous group of nanoparticles characterized by appropriate surface receptors (Table 1). Specific protein markers may be associated with different properties of the vesicles, affecting their ability to induce programmed death against different types of target cells or affecting their immune system stimulation [169,182,183,184]. Achieving the described relationships between cells through membrane vesicles secreted into the intercellular space enables an autocrine and paracrine means of intercellular communication, which favors the modification of both local and distant microenvironments [185]. The diverse load of EVs, apart from core proteins that reflect their origin and function, may also contain proteins phenotypically and physiologically identical to primary cells responsible for the secretion of specific vesicle populations, which means that they may provide important information about the pathological processes of the medical state of some individuals [186,187,188,189,190].

### 5.2. Melatonin-Dependent EVs

**Figure 2:**
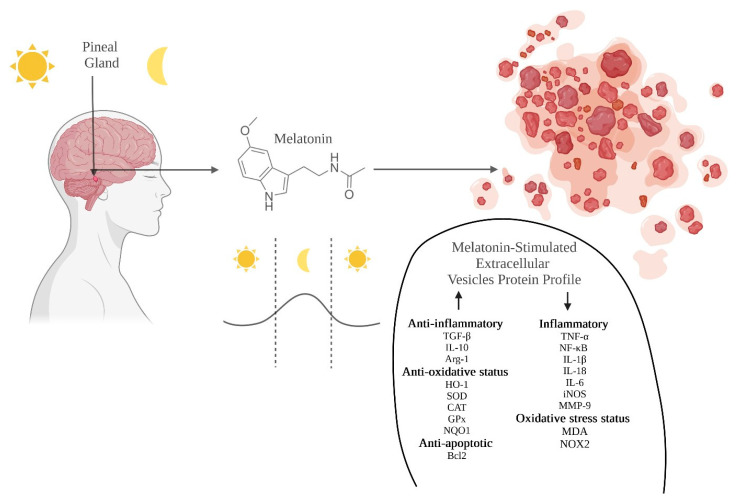
**Circadian rhythms in regulation of melatonin-dependent secretion of the EVs.** Melatonin is a ubiquitous molecule, synthesized in the pineal gland, and has myriad biological functions which primarily lead to the regulation of the endocrine circadian rhythm of the body. The presence of melatonin in the cellular environment changes the molecular composition of EVs. Melatonin is characterized by anti-inflammatory functions. The connection of EVs and melatonin represents a promising therapeutic instrument [191,192,193,194,195].

Due to the multifunctional nature of melatonin, it is considered one of the most important molecules providing hope in the dilemma concerning the connection of the unique diverse functions of EVs with their clinical application [191,192]. Melatonin is considered as the cornucopia among other neuromolecules, and its introduction into the selected populations of EVs induces modifications which have a strong protective effect on the surrounding cells (Figure 2). This is due to the ability of this biogenic amine to restore homeostasis in the body by stimulating the action of antioxidant enzymes, while having the ability to directly remove reactive oxygen and nitrogen species. This indicates the strong regulating properties of melatonin and its metabolites against the immune system, additionally showing a protective effect in diseases associated with oxidative stress [193,194,195].

It has been reported that the Toll-like receptor (TLR4)/NF-κB pathway connected with melatonin activity increases the anti-inflammatory possibilities of EVs via the stimulation of macrophage polarization. Melatonin-promoted EVs lead to the transformation of the M2 macrophage type by a phosphatase and tensin homolog deleted in the chromosome 10/Protein kinase B (PTEN)/PKB signaling pathway [196,197]. Melatonin-promoted EVs are characterized by the decreased exposition of vesicles signature cytokines, including IL-1*β*, IL-18, IL-6, and tumor necrosis factor-alpha (TNF-α), while an increase in the release of anti-inflammatory factors IL-10 and the conversion of TGF-β are observed [198,199].

Melatonin is a powerful antioxidant that scavenges various types of free radicals in body fluids and cells [200]. It has a protective effect against cellular oxidative stress, which includes anti-apoptotic actions. Melatonin-mediated EVs may play a neuroprotective role by upregulating the expression of the anti-apoptotic B-cell protein gene, which is observed in many neoplastic diseases, such as lymphomas [201,202]. The increased level of melatonin in the ECM environment modifies the biogenesis of EVs by regulating the secretion process from donor cells. Due to the relatively small size of EVs, their secretion may follow a mechanism characteristic of the secretion of low-molecular-weight metabolites, known in the literature as exocytosis. The mentioned process is based on the connection of secretory vesicles with the plasma membrane and the release of vesicle content into the extracellular space, leading to the selected proteins and lipids’ inclusion into the plasma membrane [203,204,205,206]. In the first step, melatonin activates the phosphatidylinositol 3-kinase/protein kinase B (PI3K/PKB) axis, while inhibiting the activity of glycogen synthase kinase 3 (GSK-3) [207]. During melatonin-supported exocytosis, an elevated donor cell membrane potential and increase in the elasticity and fluidity of the membrane have been observed. These symptoms are the result of the impending release of EV. There is also an increased metabolism of fatty acids in the cells responsible for extracellular secretion. Thus, the presence of melatonin in the cellular environment may result in enhanced intercellular communication, leading to increased exosome secretion [208].

Interestingly, the secretion of EVs can be supported by the process known as self-clearing the cells, described as autophagy [209]. Autophagy is considered as a regulated self-degradation process that can modify the mechanisms of exosome biogenesis in response to changes in external stimuli, related in this case to the presence of melatonin in the environment [210,211,212,213]. This is indicated primarily by reports which show that proteins responsible for the initiation and progression of cytosol and membrane autophagy take an active part in the formation and secretion of exosomes. Guo and Gil proved that the regulation of the autophagic system is based on the same signal transduction pathways as the formation of EVs [214]. The common denominator for these two, so far independent, processes, has become a conservative interaction involving the autophagy-related protein (ATG), such as ATG5 and ATG16L1 proteins [215]. Moreover, the presence of melatonin in the cellular environment directly induces autophagy by activating a number of proteins from the ATG protein family (4, 5, 7, 10, 12, 16), and the ratio of microtubule-associated protein 1A/1B-light chain (LC3) II/I is increased. In the context of EV secretion, the increased expression of these proteins enhances the fusion of multivesicular bodies (MVBs) with autophagic vacuoles and generates hybrid vesicles [216,217,218,219]. Due to the activity of specific guanosine triphosphatases (GTPases) such as Ras-related protein Rab-8A and Ras-related protein Rab-27 (Rab8a and Rab27), the release process of exosomes and autophagic contents is being carefully studied [220,221]. Thus, the presence of melatonin affects the overexpression of proteins responsible for the efficient course of autophagy, thereby changing the exosome production pathways and their content. Melatonin’s enrichment of the ECM may also provide the activation of other, alternative exosome secretory pathways, which are not observed in the melatonin-free environment [222,223].

The results of the bioinformatic analyses indicate a high correlation between the Wnt pathway proteins and melatonin-induced production of EVs. Based on the conducted analyses, the expression of paired box 2 (Pax2) and transducing-like cleavage enhancer 4 (TLE4) genes, characteristic of the course of the Wnt pathway, significantly enhances the secretion of EVs. Overexpression of these genes leads to the induction of specific intracellular signals, which then regulate the biogenesis of exosomes. However, the influence of the presence of melatonin on the alternative pathways of exosome biogenesis described above requires more extensive explanation in further studies [215,216,217,218,219,220,221,222,223].

The latest scientific reports indicate that the presence of melatonin in the environment of EVs can affect the size of exosomes, depending on the cells from which they were secreted [60,224]. The source of melatonin’s varied influence on the size of vesicles is therefore at the basis of the mechanisms of their biogenesis. For example, the presence of melatonin in bovine granulosa cells enhances the production of exosomes, but their diameter is much smaller than that of physiologically secreted exosomes. In some cases, i.e., SH-SY5Y human neuroblastoma cells, the presence of melatonin decreased the size of the vesicles, reducing it by as much as 36.23% [60,225]. Another feature of EVs influenced by the presence of melatonin in the ECM is their content, which is based primarily on mRNA, miRNAs and proteins [60]. In line with the topic of this work, the intercellular environment of anti-inflammatory macrophages enriched in melatonin induces an increase in the expression of transferring exosomes miRNA, such as miR-135b, miR-34a and miR-124, which distinguishes them from other EVs in which the above-mentioned tryptophan derivative is not observed [60,226]. Moreover, it has been shown that melatonin-stimulated EVs are characterized by increased levels of miR-4516. In the case of EVs secreted by smooth muscle, preliminary studies indicate that the presence of melatonin induces an increase in the expression of miR-204 and miR-211 in exosomes, compared to vesicles whose content was not subjected to melatonin modifications. The elevation in miR-181 in melatonin-treated exosomes is also observed in vesicles secreted by bone tissue cells, while enhancing the effect of osteogenesis. Notably, high expression of these molecules regulating the expression of exosome genes induced by the presence of melatonin may attenuate inflammation by modulating the immunoregulatory properties of EVs against target cells [60,225,226,227,228].

The presence of melatonin does not, however, affect all properties of exosomes. For example, high levels of melatonin in the cellular environment leave the unchanged tetraspanin levels of CD9, CD63, and CD81. The presence of melatonin also does not involve apoptosis regulators such as apoptosis-linked gene 2-interacting protein X (ALIX) and tumor progression genes, among which the tumor susceptibility gene 101 has been distinguished. The expression of protein markers on the surface of exosomes is extremely important in terms of the functionality of these structures. These specific surface molecules regulate internalization, immune evasion, and targeted exosome transport to target cells [60,225,226,227,228]. The issue of the level of proteins constituting the cargo of EVs, such as cellular prion protein (PrPC) or α-ketoglutarate, is different. Enrichment of the intercellular environment with melatonin affects the growth of these proteins inside the exosomes, which increases the proliferation and release of angiogenic cytokines to mitigate the inflammatory response [228,229,230].

In addition, exosomes are known to horizontally transfer melatonin between cells. Passive melatonin transport across the cell membrane is possible due to its lipophilic layer and passive membrane diffusion. Researchers are extremely interested in the mechanism of transfer of the high-affinity G protein-coupled receptors named MT1 melatonin receptor by internalization and endocytic transport. When melatonin signaling is connected to MT1, Rab5 supports the relocation of the internalized MT1 to early endosomes. MT1-carrying endosomes can cross the plasma membrane through the activity of other GTPases [60,220,221,222,223,224,225,226,227,228,229,230,231,232,233].

## 6. Conclusions

The development and complications of cardiovascular diseases are largely based on pro-inflammatory cytokine-signaling pathways. Dysfunction and activation of the endothelium leading to inflammation occur in response to the induction of ROS production by a myriad of proinflammatory cytokines. Due to its antioxidant properties, melatonin is a pharmaceutical that specifically targets the molecular and signaling pathways involved in the pathophysiology of CVDs, which has been demonstrated in the example of TGF-β. TGF-β is a cytokine that causes the growth and proliferation of many types of cells, and thus affects the increased production of EV populations. Despite the existence of many papers describing TGF-β-dependent vesicle secretion and melatonin-stimulated follicular secretion, there is still a lack of data confirming the synergy of these two factors in the secretion process.

## Figures and Tables

**Table 1:** Detail classification and characteristics of EV populations (modified based on 186–190).

Vesicle Type						
	Exosomes	Microvesicles	Apoptotic Bodies	Oncosomes	Exophers	Migrasome
Morphology (by TEM)	Cup shape	Irregular shape	Oval shape	Heterogeneous	Quasi-Spherical Bodies	Pomegranate-like structures
Diameter (nm)	30–200	50–1000	50–5000	1000–10,000	+/−4000	500–2000
Density (g/mL)	1.13–1.19	1.04–1.07	1.16–1.28	N/A	N/A	N/A
Biogenesis	ESCRT endocytic pathway Ceramide-dependent multivesicular bodies	Cell Surface; Plasma membrane shedding	Cell Surface; Release by cell fragmentation during shrinkage caused to cell death (apoptosis)	Plasma membrane blebbing from cells	Budding out of cells into the extracellular space	Retraction fibers; Migracytosis
Enriched Markers	CD63 CD9 CD81 CD82 Hsp60 Hsp70 Hsp90 ALIX TSG101 PDCD6IP LAMP1 Flotillin-1 Rab27 ESCRT proteins	CD14 CD31 CD34 CD51 CD62E CD40 LL-37 HMGB1 ARF6 Integrin β1 VAMP3 ADAM10 NOTCH2	Trp-BODIPY cyclic peptide Annexin V C3b gp96 PANX1 Caspase-3 Caspase-7 VDAC1	CD63 CD9 CD81 Cytokeratin-18 EGFR AKT1 Cav-1 ARF6 CK18 MMP-2 MMP-9 eEF1γ αV-integrin MDH GPI-Aps	MAP2 β-III tubulin tau protein	Tspan-4 Tspan-7 Integrinα5β1 NDST1
Molecular Cargo	Lipid Proteins Nucleic acids Non-coding RNAs MHC molecules	Lipid Proteins Nucleic acids Non-coding RNAs	Nuclear fractions	Protein Nucleic acids Non-coding RNAs	Cell organelles	
Processes	Intercellular communication via paracrine, autocrine, endocrine, and cell-to-cell contact signaling					
Detection	Flow Cytometry ELISA Cryo-EM TEM SEM WB AFM DLS RPS Proteomics			TEM SEM IF WB		

## References

[1] Humphrey J.D., Schwartz M.A. (2021). Vascular Mechanobiology: Homeostasis, Adaptation, and Disease. Annu. Rev. Biomed. Eng..

[2] Xu S., Lyu Q.R., Ilyas I., Tian X.Y., Weng J. (2022). Vascular homeostasis in atherosclerosis: A holistic overview. Front. Immunol..

[3] Huwiler A., Pfeilschifter J. (2021). Recuperation of vascular homeostasis. Circ. Res..

[4] Pober J.S., Sessa W.C. (2014). Inflammation and the blood microvascular system. Cold Spring Harb. Perspect. Biol..

[5] Arnout J., Hoylaerts M.F., Lijnen H.R. (2006). Haemostasis. Handb. Exp. Pharmacol..

[6] Schnoor M., Alcaide P., Voisin M.B., van Buul J.D. (2015). Crossing the Vascular Wall: Common and Unique Mechanisms Exploited by Different Leukocyte Subsets during Extravasation. Mediat. Inflamm..

[7] Claesson-Welsh L., Dejana E., McDonald D.M. (2021). Permeability of the Endothelial Barrier: Identifying and Reconciling Controversies. Trends Mol. Med..

[8] Hellenthal K.E.M., Brabenec L., Wagner N.M. (2022). Regulation and Dysregulation of Endothelial Permeability during Systemic Inflammation. Cells.

[9] Shah P.K., Lecis D. (2019). Inflammation in atherosclerotic cardiovascular disease. F1000Research.

[10] Ruparelia N., Chai J.T., Fisher E.A., Choudhury R.P. (2017). Inflammatory processes in cardiovascular disease: A route to targeted therapies. Nat. Rev. Cardiol..

[11] Hansson G.K. (2005). Inflammation, atherosclerosis, and coronary artery disease. N. Engl. J. Med..

[12] Sokol C.L., Luster A.D. (2015). The chemokine system in innate immunity. Cold Spring Harb. Perspect. Biol..

[13] Roy I., Evans D.B., Dwinell M.B. (2014). Chemokines and chemokine receptors: Update on utility and challenges for the clinician. Surgery.

[14] Hughes C.E., Nibbs R.J.B. (2018). A guide to chemokines and their receptors. FEBS J..

[15] Muller W.A. (2013). Getting leukocytes to the site of inflammation. Vet. Pathol..

[16] Johnson L.A., Clasper S., Holt A.P., Lalor P.F., Baban D., Jackson D.G. (2006). An inflammation-induced mechanism for leukocyte transmigration across lymphatic vessel endothelium. J. Exp. Med..

[17] Langer H.F., Chavakis T. (2009). Leukocyte-endothelial interactions in inflammation. J. Cell. Mol. Med..

[18] Harjunpää H., Llort Asens M., Guenther C., Fagerholm S.C. (2019). Cell Adhesion Molecules and Their Roles and Regulation in the Immune and Tumor Microenvironment. Front. Immunol..

[19] Salminen A.T., Allahyari Z., Gholizadeh S., McCloskey M.C., Ajalik R., Cottle R.N., Gaborski T.R., McGrath J.L. (2020). *NF* Studies of Transendothelial Migration for Biological and Drug Discovery. Front. Med. Technol..

[20] Sun L., Ye R.D. (2012). Role of G protein-coupled receptors in inflammation. Acta Pharmacol. Sin..

[21] Mitroulis I., Alexaki V.I., Kourtzelis I., Ziogas A., Hajishengallis G., Chavakis T. (2015). Leukocyte integrins: Role in leukocyte recruitment and as therapeutic targets in inflammatory disease. Pharmacol. Ther..

[22] Frank P.G., Lisanti M.P. (2008). ICAM-1: Role in inflammation and in the regulation of vascular permeability. Am. J. Physiol. Heart Circ. Physiol..

[23] Filippi M.D. (2016). Mechanism of Diapedesis: Importance of the Transcellular Route. Adv. Immunol..

[24] Barthel S.R., Gavino J.D., Descheny L., Dimitroff C.J. (2007). Targeting selectins and selectin ligands in inflammation and cancer. Expert Opin. Ther. Targets.

[25] Hyun Y.M., Lefort C.T., Kim M. (2009). Leukocyte integrins and their ligand interactions. Immunol. Res..

[26] Vazquez M.I., Catalan-Dibene J., Zlotnik A. (2015). B cells responses and cytokine production are regulated by their immune microenvironment. Cytokine.

[27] McEver R.P. (2015). Selectins: Initiators of leucocyte adhesion and signalling at the vascular wall. Cardiovasc. Res..

[28] Moser B., Willimann K. (2004). Chemokines: Role in inflammation and immune surveillance. Ann. Rheum. Dis..

[29] Kraaijeveld A.O., de Jager S.C., van Berkel T.J., Biessen E.A., Jukema J.W. (2007). Chemokines and atherosclerotic plaque progression: Towards therapeutic targeting?. Curr. Pharm. Des..

[30] Lu X., Wang Z., Ye D., Feng Y., Liu M., Xu Y., Wang M., Zhang J., Liu J., Zhao M. (2022). The Role of CXC Chemokines in Cardiovascular Diseases. Front. Pharmacol..

[31] Cui M.Z. (2011). Lysophosphatidic acid effects on atherosclerosis and thrombosis. Clin. Lipidol..

[32] Gencer S., Evans B.R., van der Vorst E.P.C., Döring Y., Weber C. (2021). Inflammatory Chemokines in Atherosclerosis. Cells.

[33] Cambier S., Gouwy M., Proost P. (2023). The chemokines CXCL8 and CXCL12: Molecular and functional properties, role in disease and efforts towards pharmacological intervention. Cell. Mol. Immunol..

[34] Weber C., Meiler S., Döring Y., Koch M., Drechsler M., Megens R.T., Rowinska Z., Bidzhekov K., Fecher C., Ribechini E. (2011). CCL17-expressing dendritic cells drive atherosclerosis by restraining regulatory T cell homeostasis in mice. J. Clin. Investig..

[35] Feng G., Bajpai G., Ma P., Koenig A., Bredemeyer A., Lokshina I., Lai L., Förster I., Leuschner F., Kreisel D. (2022). CCL17 Aggravates Myocardial Injury by Suppressing Recruitment of Regulatory T Cells. Circulation.

[36] Taverna S., Amodeo V., Saieva L., Russo A., Giallombardo M., De Leo G., Alessandro R. (2014). Exosomal shuttling of miR-126 in endothelial cells modulates adhesive and migratory abilities of chronic myelogenous leukemia cells. Mol. Cancer.

[37] Bassand K., Metzinger L., Naïm M., Mouhoubi N., Haddad O., Assoun V., Zaïdi N., Sainte-Catherine O., Butt A., Guyot E. (2021). miR-126-3p is essential for CXCL12-induced angiogenesis. J. Cell. Mol. Med..

[38] Zheng J., Yang M., Shao J., Miao Y., Han J., Du J. (2013). Chemokine receptor CX3CR1 contributes to macrophage survival in tumor metastasis. Mol. Cancer.

[39] Rousselle A., Qadri F., Leukel L., Yilmaz R., Fontaine J.F., Sihn G., Bader M., Ahluwalia A., Duchene J. (2013). CXCL5 limits macrophage foam cell formation in atherosclerosis. J. Clin. Investig..

[40] Surmi B.K., Hasty A.H. (2010). The role of chemokines in recruitment of immune cells to the artery wall and adipose tissue. Vascul. Pharmacol..

[41] Van der Vorst E.P., Döring Y., Weber C. (2015). Chemokines and their receptors in Atherosclerosis. J. Mol. Med..

[42] Peng D., Fu M., Wang M., Wei Y., Wei X. (2022). Targeting TGF-β signal transduction for fibrosis and cancer therapy. Mol. Cancer.

[43] Siegel P.M., Massagué J. (2003). Cytostatic and apoptotic actions of TGF-β in homeostasis and cancer. Nat. Rev. Cancer.

[44] Zhao H., Wei J., Sun J. (2020). Roles of TGF-β signaling pathway in tumor microenvirionment and cancer therapy. Int. Immunopharmacol..

[45] Kobayashi M., Fujiwara K., Takahashi K., Yoshioka Y., Ochiya T., Podyma-Inoue K.A., Watabe T. (2022). Transforming growth factor-β-induced secretion of extracellular vesicles from oral cancer cells evokes endothelial barrier instability *via* endothelial-mesenchymal transition. Inflamm. Regen..

[46] Goumans M.J., Ten Dijke P. (2018). TGF-β Signaling in Control of Cardiovascular Function. Cold Spring Harb. Perspect. Biol..

[47] Dagher Z., Gerhardinger C., Vaz J., Goodridge M., Tecilazich F., Lorenzi M. (2017). The Increased Transforming Growth Factor-β Signaling Induced by Diabetes Protects Retinal Vessels. Am. J. Pathol..

[48] Tobeiha M., Jafari A., Fadaei S., Mirazimi S.M.A., Dashti F., Amiri A., Khan H., Asemi Z., Reiter R.J., Hamblin M.R. (2022). Evidence for the Benefits of Melatonin in Cardiovascular Disease. Front. Cardiovasc. Med..

[49] Baburina Y., Lomovsky A., Krestinina O. (2021). Melatonin as a Potential Multitherapeutic Agent. J. Pers. Med..

[50] Lee F.Y., Sun C.K., Sung P.H., Chen K.H., Chua S., Sheu J.J., Chung S.Y., Chai H.T., Chen Y.L., Huang T.H. (2018). Daily melatonin protects the endothelial lineage and functional integrity against the aging process, oxidative stress, and toxic environment and restores blood flow in critical limb ischemia area in mice. J. Pineal Res..

[51] Galano A., Tan D.X., Reiter R.J. (2018). Melatonin: A Versatile Protector against Oxidative DNA Damage. Molecules.

[52] Reiter R.J., Mayo J.C., Tan D.X., Sainz R.M., Alatorre-Jimenez M., Qin L. (2016). Melatonin as an antioxidant: Under promises but over delivers. J. Pineal Res..

[53] Batty M., Bennett M.R., Yu E. (2022). The Role of Oxidative Stress in Atherosclerosis. Cells.

[54] Minich D.M., Henning M., Darley C., Fahoum M., Schuler C.B., Frame J. (2022). Is Melatonin the “Next Vitamin D”?: A Review of Emerging Science, Clinical Uses, Safety, and Dietary Supplements. Nutrients.

[55] Bonowicz K., Mikołajczyk K., Faisal I., Qamar M., Steinbrink K., Kleszczyński K., Grzanka A., Gagat M. (2022). Mechanism of Extracellular Vesicle Secretion Associated with TGF-β-Dependent Inflammatory Response in the Tumor Microenvironment. Int. J. Mol. Sci..

[56] Phillips W., Willms E., Hill A.F. (2021). Understanding extracellular vesicle and nanoparticle heterogeneity: Novel methods and considerations. Proteomics.

[57] Holcar M., Kandušer M., Lenassi M. (2021). Blood Nanoparticles–Influence on Extracellular Vesicle Isolation and Characterization. Front. Pharmacol..

[58] Di Bella M.A. (2022). Overview and Update on Extracellular Vesicles: Considerations on Exosomes and Their Application in Modern Medicine. Biology.

[59] Ludwig N., Yerneni S.S., Azambuja J.H., Pietrowska M., Widłak P., Hinck C.S., Głuszko A., Szczepański M.J., Kärmer T., Kallinger I. (2022). TGFβ+ small extracellular vesicles from head and neck squamous cell carcinoma cells reprogram macrophages towards a pro-angiogenic phenotype. J. Extracell. Vesicles.

[60] Amini H., Rezabakhsh A., Heidarzadeh M., Hassanpour M., Hashemzadeh S., Ghaderi S., Sokullu E., Rahbarghazi R., Reiter R.J. (2021). An Examination of the Putative Role of Melatonin in Exosome Biogenesis. Front. Cell. Dev. Biol..

[61] Abels E.R., Breakefield X.O. (2016). Introduction to Extracellular Vesicles: Biogenesis, RNA Cargo Selection, Content, Release, and Uptake. Cell. Mol. Neurobiol..

[62] Wolf D., Ley K. (2019). Immunity and Inflammation in Atherosclerosis. Circ. Res..

[63] Björkegren J.L.M., Lusis A.J. (2022). Atherosclerosis: Recent developments. Cell.

[64] Krüger-Genge A., Blocki A., Franke R.P., Jung F. (2019). Vascular Endothelial Cell Biology: An Update. Int. J. Mol. Sci..

[65] Campinho P., Vilfan A., Vermot J. (2020). Blood Flow Forces in Shaping the Vascular System: A Focus on Endothelial Cell Behavior. Front. Physiol..

[66] Dhawan S.S., Avati Nanjundappa R.P., Branch J.R., Taylor W.R., Quyyumi A.A., Jo H., McDaniel M.C., Suo J., Giddens D., Samady H. (2010). Shear stress and plaque development. Expert Rev. Cardiovasc. Ther..

[67] Siasos G., Sara J.D., Zaromytidou M., Park K.H., Coskun A.U., Lerman L.O., Oikonomou E., Maynard C.C., Fotiadis D., Stefanou K. (2018). Local Low Shear Stress and Endothelial Dysfunction in Patients With Nonobstructive Coronary Atherosclerosis. J. Am. Coll. Cardiol..

[68] Theofilis P., Sagris M., Oikonomou E., Antonopoulos A.S., Siasos G., Tsioufis C., Tousoulis D. (2021). Inflammatory Mechanisms Contributing to Endothelial Dysfunction. Biomedicines.

[69] Park K.H., Park W.J. (2015). Endothelial Dysfunction: Clinical Implications in Cardiovascular Disease and Therapeutic Approaches. J. Korean Med. Sci..

[70] Nappi F., Fiore A., Masiglat J., Cavuoti T., Romandini M., Nappi P., Avtaar Singh S.S., Couetil J.P. (2022). Endothelium-Derived Relaxing Factors and Endothelial Function: A Systematic Review. Biomedicines.

[71] Pan S. (2009). Molecular mechanisms responsible for the atheroprotective effects of laminar shear stress. Antioxid. Redox Signal..

[72] Yuyun M.F., Ng L.L., Ng G.A. (2018). Endothelial dysfunction, endothelial nitric oxide bioavailability, tetrahydrobiopterin, and 5-methyltetrahydrofolate in cardiovascular disease. Where are we with therapy?. Microvasc. Res..

[73] Berenji Ardestani S., Eftedal I., Pedersen M. (2020). Endothelial dysfunction in small arteries and early signs of atherosclerosis in ApoE knockout rats. Sci. Rep..

[74] Mudau M., Genis A., Lochner A., Strijdom H. (2012). Endothelial dysfunction: The early predictor of atherosclerosis. Cardiovasc. J. Afr..

[75] Guipaud O., Jaillet C., Clément-Colmou K., François A., Supiot S., Milliat F. (2018). The importance of the vascular endothelial barrier in the immune-inflammatory response induced by radiotherapy. Br. J. Radiol..

[76] Kumar Rajendran N., George B.P., Chandran R., Tynga I.M., Houreld N., Abrahamse H. (2019). The Influence of Light on Reactive Oxygen Species and NF-κB in Disease Progression. Antioxidants.

[77] Lingappan K. (2018). NF-κB in Oxidative Stress. Curr. Opin. Toxicol..

[78] Zhang C. (2008). The role of inflammatory cytokines in endothelial dysfunction. Basic Res. Cardiol..

[79] Zhang H., Park Y., Wu J., Chen X., Lee S., Yang J., Dellsperger K.C., Zhang C. (2009). Role of TNF-alpha in vascular dysfunction. Clin. Sci..

[80] Blaser H., Dostert C., Mak T.W., Brenner D. (2016). TNF and ROS Crosstalk in Inflammation. Trends Cell. Biol..

[81] Ende G., Poitz D.M., Wiedemann E., Augstein A., Friedrichs J., Giebe S., Weinert S., Werner C., Strasser R.H., Jellinghaus S. (2014). TNF-α-mediated adhesion of monocytes to endothelial cells-The role of ephrinA1. J. Mol. Cell. Cardiol..

[82] Deshmane S.L., Kremlev S., Amini S., Sawaya B.E. (2009). Monocyte chemoattractant protein-1 (MCP-1): An overview. J. Interferon. Cytokine Res..

[83] Kang H., Li X., Xiong K., Song Z., Tian J., Wen Y., Sun A., Deng X. (2021). The Entry and Egress of Monocytes in Atherosclerosis: A Biochemical and Biomechanical Driven Process. Cardiovasc. Ther..

[84] Bobryshev Y.V., Ivanova E.A., Chistiakov D.A., Nikiforov N.G., Orekhov A.N. (2016). Macrophages and Their Role in Atherosclerosis: Pathophysiology and Transcriptome Analysis. Biomed. Res. Int..

[85] Moore K.J., Sheedy F.J., Fisher E.A. (2013). Macrophages in atherosclerosis: A dynamic balance. Nat. Rev. Immunol..

[86] Persson J., Nilsson J., Lindholm M.W. (2006). Cytokine response to lipoprotein lipid loading in human monocyte-derived macrophages. Lipids Health Dis..

[87] Sun L., Zhang W., Zhao Y., Wang F., Liu S., Liu L., Zhao L., Lu W., Li M., Xu Y. (2020). Dendritic Cells and T Cells, Partners in Atherogenesis and the Translating Road Ahead. Front. Immunol..

[88] Ilhan F., Kalkanli S.T. (2015). Atherosclerosis and the role of immune cells. World J. Clin. Cases.

[89] Yousaf H., Khan M.I.U., Ali I., Munir M.U., Lee K.Y. (2023). Emerging role of macrophages in non-infectious diseases: An update. Biomed. Pharmacother..

[90] Boehncke W.H., Schön M.P., Girolomoni G., Griffiths C., Bos J.D., Thestrup-Pedersen K., Cavani A., Nestle F., Bonish B.K., Campbell J.J. (2005). Leukocyte extravasation as a target for anti-inflammatory therapy—Which molecule to choose?. Exp. Dermatol..

[91] Bennett M.R., Sinha S., Owens G.K. (2016). Vascular Smooth Muscle Cells in Atherosclerosis. Circ. Res..

[92] Hu D., Yin C., Luo S., Habenicht A.J.R., Mohanta S.K. (2019). Vascular Smooth Muscle Cells Contribute to Atherosclerosis Immunity. Front. Immunol..

[93] Jaminon A., Reesink K., Kroon A., Schurgers L. (2019). The Role of Vascular Smooth Muscle Cells in Arterial Remodeling: Focus on Calcification-Related Processes. Int. J. Mol. Sci..

[94] Triggle C.R., Samuel S.M., Ravishankar S., Marei I., Arunachalam G., Ding H. (2012). The endothelium: Influencing vascular smooth muscle in many ways. Can. J. Physiol. Pharmacol..

[95] Sorokin V., Vickneson K., Kofidis T., Woo C.C., Lin X.Y., Foo R., Shanahan C.M. (2020). Role of Vascular Smooth Muscle Cell Plasticity and Interactions in Vessel Wall Inflammation. Front. Immunol..

[96] Li H., Bai S., Ao Q., Wang X., Tian X., Li X., Tong H., Hou W., Fan J. (2018). Modulation of Immune-Inflammatory Responses in Abdominal Aortic Aneurysm: Emerging Molecular Targets. J. Immunol. Res..

[97] Chen J., Chang R. (2022). Association of TGF-β Canonical Signaling-Related Core Genes With Aortic Aneurysms and Aortic Dissections. Front. Pharmacol..

[98] Tsuda T. (2018). Extracellular Interactions between Fibulins and Transforming Growth Factor (TGF)-β in Physiological and Pathological Conditions. Int. J. Mol. Sci..

[99] IJpma A., te Riet L., van de Luijtgaarden K.M., van Heijningen P.M., Burger J., Majoor-Krakauer D., Rouwet E.V., Essers J., Verhagen H.J.M., van der Pluijm I. (2019). Inflammation and TGF-β Signaling Differ between Abdominal Aneurysms and Occlusive Disease. J. Cardiovasc. Dev. Dis..

[100] Xu X., Zheng L., Yuan Q., Zhen G., Crane J.L., Zhou X., Cao X. (2018). Transforming growth factor-β in stem cells and tissue homeostasis. Bone Res..

[101] Serralheiro P., Soares A., Costa Almeida C.M., Verde I. (2017). TGF-β1 in Vascular Wall Pathology: Unraveling Chronic Venous Insufficiency Pathophysiology. Int. J. Mol. Sci..

[102] Zhang Y., Alexander P.B., Wang X.F. (2017). TGF-β Family Signaling in the Control of Cell Proliferation and Survival. Cold Spring Harb. Perspect. Biol..

[103] Tzavlaki K., Moustakas A. (2020). TGF-β Signaling. Biomolecules.

[104] Robertson I.B., Horiguchi M., Zilberberg L., Dabovic B., Hadjiolova K., Rifkin D.B. (2015). Latent TGF-β-binding proteins. Matrix Biol..

[105] Rifkin D., Sachan N., Singh K., Sauber E., Tellides G., Ramirez F. (2022). The role of LTBPs in TGF β signaling. Dev. Dyn..

[106] Robertson I.B., Rifkin D.B. (2016). Regulation of the Bioavailability of TGF-β and TGF-β-Related Proteins. Cold Spring Harb. Perspect. Biol..

[107] Li Y., Fan W., Link F., Wang S., Dooley S. (2021). Transforming growth factor β latency: A mechanism of cytokine storage and signalling regulation in liver homeostasis and disease. JHEP Rep..

[108] Walton K.L., Makanji Y., Chen J., Wilce M.C., Chan K.L., Robertson D.M., Harrison C.A. (2010). Two distinct regions of latency-associated peptide coordinate stability of the latent transforming growth factor-β1 complex. J. Biol. Chem..

[109] Taylor A.W. (2009). Review of the activation of TGF-β in immunity. J. Leukoc. Biol..

[110] Huang F., Chen Y.G. (2012). Regulation of TGF-β receptor activity. Cell Biosci..

[111] Yakymovych I., Yakymovych M., Hamidi A., Landström M., Heldin C.H. (2022). The type II TGF-β receptor phosphorylates Tyr182 in the type I receptor to activate downstream Src signaling. Sci. Signal..

[112] Huynh L.K., Hipolito C.J., Ten Dijke P. (2019). A Perspective on the Development of TGF-β Inhibitors for Cancer Treatment. Biomolecules.

[113] Haque S., Morris J.C. (2017). Transforming growth factor-β: A therapeutic target for cancer. Hum. Vaccines Immunother..

[114] Heldin C.H., Moustakas A. (2016). Signaling Receptors for TGF-β Family Members. Cold Spring Harb. Perspect. Biol..

[115] Zakrzewski P.K. (2021). Canonical TGFβ Signaling and Its Contribution to Endometrial Cancer Development and Progression-Underestimated Target of Anticancer Strategies. J. Clin. Med..

[116] Zou M.L., Chen Z.H., Teng Y.Y., Liu S.Y., Jia Y., Zhang K.W., Sun Z.L., Wu J.J., Yuan Z.D., Feng Y. (2021). The Smad Dependent TGF-β and BMP Signaling Pathway in Bone Remodeling and Therapies. Front. Mol. Biosci..

[117] Hiepen C., Mendez P.L., Knaus P. (2020). It Takes Two to Tango: Endothelial TGFβ/BMP Signaling Crosstalk with Mechanobiology. Cells.

[118] Pierreux C.E., Nicolás F.J., Hill C.S. (2000). Transforming growth factor β-independent shuttling of Smad4 between the cytoplasm and nucleus. Mol. Cell. Biol..

[119] Pardali E., Ten Dijke P. (2012). TGFβ signaling and cardiovascular diseases. Int. J. Biol. Sci..

[120] Mulder K.M. (2000). Role of Ras and Mapks in TGFβ signaling. Cytokine Growth Factor Rev..

[121] Kim S.I., Kwak J.H., Na H.J., Kim J.K., Ding Y., Choi M.E. (2009). Transforming growth factor-β (TGF-β1) activates TAK1 *via* TAB1-mediated autophosphorylation, independent of TGF-β receptor kinase activity in mesangial cells. J. Biol. Chem..

[122] Grusch M., Petz M., Metzner T., Oztürk D., Schneller D., Mikulits W. (2010). The crosstalk of RAS with the TGF-β family during carcinoma progression and its implications for targeted cancer therapy. Curr. Cancer Drug Targets.

[123] Xu L. (2006). Regulation of Smad activities. Biochim. Biophys. Acta.

[124] Bhattacharyya S., Chen S.J., Wu M., Warner-Blankenship M., Ning H., Lakos G., Mori Y., Chang E., Nihijima C., Takehara K. (2008). Smad-independent transforming growth factor-β regulation of early growth response-1 and sustained expression in fibrosis: Implications for scleroderma. Am. J. Pathol..

[125] Perlman R., Schiemann W.P., Brooks M.W., Lodish H.F., Weinberg R.A. (2001). TGF-β-induced apoptosis is mediated by the adapter protein Daxx that facilitates JNK activation. Nat. Cell Biol..

[126] Chen C., Lei W., Chen W., Zhong J., Gao X., Li B., Wang H., Huang C. (2014). Serum TGF-β1 and SMAD3 levels are closely associated with coronary artery disease. BMC Cardiovasc. Disord..

[127] Kulach A., Dabek J., Wilczok T., Gasior Z. (2010). Changes in transforming growth factor β and its receptors’ mRNA expression in monocytes from patients with acute coronary syndromes. Arch. Med. Sci..

[128] Wu L., Derynck R. (2009). Essential role of TGF-β signaling in glucose-induced cell hypertrophy. Dev. Cell.

[129] Fleisch M.C., Maxwell C.A., Barcellos-Hoff M.H. (2006). The pleiotropic roles of transforming growth factor beta in homeostasis and carcinogenesis of endocrine organs. Endocr. Relat. Cancer.

[130] Coomes S.M., Moore B.B. (2010). Pleiotropic effects of transforming growth factor-β in hematopoietic stem-cell transplantation. Transplantation.

[131] Low E.L., Baker A.H., Bradshaw A.C. (2019). TGFβ, smooth muscle cells and coronary artery disease: A review. Cell. Signal..

[132] Chen P.Y., Qin L., Li G., Wang Z., Dahlman J.E., Malagon-Lopez J., Gujja S., Cilfone N.A., Kauffman K.J., Sun L. (2019). Endothelial TGF-β signalling drives vascular inflammation and atherosclerosis. Nat. Metab..

[133] Krstic J., Santibanez J.F. (2014). Transforming growth factor-beta and matrix metalloproteinases: Functional interactions in tumor stroma-infiltrating myeloid cells. Sci. World J..

[134] Vasconcelos D.B., Falcão L.F.M., da Ponte L.C.T., Silva C.C., Martins L.C., Nunes B.T.D., Martins Filho A.J., Franco E.C.S., Duarte M.I.S., Sousa J.R.d. (2022). New Insights into the Mechanism of Immune-Mediated Tissue Injury in Yellow Fever: The Role of Immunopathological and Endothelial Alterations in the Human Lung Parenchyma. Viruses.

[135] Figarella-Branger D., Civatte M., Bartoli C., Pellissier J.F. (2003). Cytokines, chemokines, and cell adhesion molecules in inflammatory myopathies. Muscle Nerve.

[136] Wang S., Zhang Q., Wang Y., You B., Meng Q., Zhang S., Li X., Ge Z. (2018). Transforming Growth Factor β1 (TGF-β1) Appears to Promote Coronary Artery Disease by Upregulating Sphingosine Kinase 1 (SPHK1) and Further Upregulating Its Downstream TIMP-1. Med. Sci. Monit..

[137] Gao Y., Ma L., Bai C., Zhang X., Yang W. (2019). Melatonin promotes self-renewal and nestin expression in neural stem cells from the retina. Histol. Histopathol..

[138] Dubocovich M.L., Delagrange P., Krause D.N., Sugden D., Cardinali D.P., Olcese J. (2010). International Union of Basic and Clinical Pharmacology. LXXV. Nomenclature, classification, and pharmacology of G protein-coupled melatonin receptors. Pharmacol. Rev..

[139] Doghramji K. (2007). Melatonin and its receptors: A new class of sleep-promoting agents. J. Clin. Sleep Med..

[140] Agez L., Laurent V., Guerrero H.Y., Pévet P., Masson-Pévet M., Gauer F. (2009). Endogenous melatonin provides an effective circadian message to both the suprachiasmatic nuclei and the pars tuberalis of the rat. J. Pineal Res..

[141] Blume C., Garbazza C., Spitschan M. (2019). Effects of light on human circadian rhythms, sleep and mood. Somnologie.

[142] Betti L., Palego L., Demontis G.C., Miraglia F., Giannaccini G. (2019). Hydroxyindole-O-methyltransferase (HIOMT) activity in the retina of melatonin-proficient mice. Heliyon.

[143] Mannino G., Pernici C., Serio G., Gentile C., Bertea C.M. (2021). Melatonin and Phytomelatonin: Chemistry, Biosynthesis, Metabolism, Distribution and Bioactivity in Plants and Animals-An Overview. Int. J. Mol. Sci..

[144] Srinivasan V., Spence D.W., Pandi-Perumal S.R., Brown G.M., Cardinali D.P. (2011). Melatonin in mitochondrial dysfunction and related disorders. Int. J. Alzheimers Dis..

[145] Slominski A.T., Zmijewski M.A., Semak I., Kim T.K., Janjetovic Z., Slominski R.M., Zmijewski J.W. (2017). Melatonin, mitochondria, and the skin. Cell. Mol. Life Sci..

[146] Bhattacharyya A., Chattopadhyay R., Mitra S., Crowe S.E. (2014). Oxidative stress: An essential factor in the pathogenesis of gastrointestinal mucosal diseases. Physiol. Rev..

[147] Mayo J.C., Sainz R.M., González-Menéndez P., Hevia D., Cernuda-Cernuda R. (2017). Melatonin transport into mitochondria. Cell. Mol. Life Sci..

[148] Sunyer-Figueres M., Vázquez J., Mas A., Torija M.J., Beltran G. (2020). Transcriptomic Insights into the Effect of Melatonin in Saccharomyces cerevisiae in the Presence and Absence of Oxidative Stress. Antioxidants.

[149] Hardeland R. (2017). Taxon- and Site-Specific Melatonin Catabolism. Molecules.

[150] Vanecek J. (1998). Cellular mechanisms of melatonin action. Physiol. Rev..

[151] Alves E., Bartlett P.J., Garcia C.R., Thomas A.P. (2011). Melatonin and IP3-induced Ca2+ release from intracellular stores in the malaria parasite Plasmodium falciparum within infected red blood cells. J. Biol. Chem..

[152] Ma H., Kang J., Fan W., He H., Huang F. (2021). ROR: Nuclear Receptor for Melatonin or Not?. Molecules.

[153] Kopustinskiene D.M., Bernatoniene J. (2021). Molecular Mechanisms of Melatonin-Mediated Cell Protection and Signaling in Health and Disease. Pharmaceutics.

[154] Besag F.M.C., Vasey M.J., Lao K.S.J., Wong I.C.K. (2019). Adverse Events Associated with Melatonin for the Treatment of Primary or Secondary Sleep Disorders: A Systematic Review. CNS Drugs.

[155] Foley H.M., Steel A.E. (2019). Adverse events associated with oral administration of melatonin: A critical systematic review of clinical evidence. Complement. Ther. Med..

[156] Liu R.M., Desai L.P. (2015). Reciprocal regulation of TGF-β and reactive oxygen species: A perverse cycle for fibrosis. Redox Biol..

[157] Arioz B.I., Tastan B., Tarakcioglu E., Tufekci K.U., Olcum M., Ersoy N., Bagriyanik A., Genc K., Genc S. (2019). Melatonin Attenuates LPS-Induced Acute Depressive-Like Behaviors and Microglial NLRP3 Inflammasome Activation Through the SIRT1/Nrf2 Pathway. Front. Immunol..

[158] Xu J., Lamouille S., Derynck R. (2009). TGF-β-induced epithelial to mesenchymal transition. Cell Res..

[159] Kim J.Y., Park J.H., Jeon E.J., Leem J., Park K.K. (2020). Melatonin Prevents Transforming Growth Factor-β1-Stimulated Transdifferentiation of Renal Interstitial Fibroblasts to Myofibroblasts by Suppressing Reactive Oxygen Species-Dependent Mechanisms. Antioxidants.

[160] Chung J., Huda M.N., Shin Y., Han S., Akter S., Kang I., Ha J., Choe W., Choi T.G., Kim S.S. (2021). Correlation between Oxidative Stress and Transforming Growth Factor-Beta in Cancers. Int. J. Mol. Sci..

[161] Edlund S., Bu S., Schuster N., Aspenström P., Heuchel R., Heldin N.E., ten Dijke P., Heldin C.H., Landström M. (2003). Transforming growth factor-β1 (TGF-β)-induced apoptosis of prostate cancer cells involves Smad7-dependent activation of p38 by TGF-β-activated kinase 1 and mitogen-activated protein kinase kinase 3. Biochim. Biophys. Acta (BBA)-Mol. Biol. Cell.

[162] Qin R., Zhao Q., Han B., Zhu H.P., Peng C., Zhan G., Huang W. (2022). Indole-Based Small Molecules as Potential Therapeutic Agents for the Treatment of Fibrosis. Front. Pharmacol..

[163] Xiong X.-C., Zhu Y., Ge R., Liu L.-F., Yuan W. (2015). Effect of Melatonin on the Extracellular-Regulated Kinase Signal Pathway Activation and Human Osteoblastic Cell Line hFOB 1.19 Proliferation. Int. J. Mol. Sci..

[164] Emet M., Ozcan H., Ozel L., Yayla M., Halici Z., Hacimuftuoglu A. (2016). A Review of Melatonin, Its Receptors and Drugs. Eurasian J. Med..

[165] Fan Z., Qi X., Yang W., Xia L., Wu Y. (2020). Melatonin Ameliorates Renal Fibrosis Through the Inhibition of NF-κB and TGF-β1/Smad3 Pathways in db/db Diabetic Mice. Arch. Med. Res..

[166] Griggs L.A., Hassan N.T., Malik R.S., Griffin B.P., Martinez B.A., Elmore L.W., Lemmon C.A. (2017). Fibronectin fibrils regulate TGF-β1-induced Epithelial-Mesenchymal Transition. Matrix Biol..

[167] Che H., Wang Y., Li H., Li Y., Sahil A., Lv J., Liu Y., Yang Z., Dong R., Xue H. (2020). Melatonin alleviates cardiac fibrosis *via* inhibiting lncRNA MALAT1/miR-141-mediated NLRP3 inflammasome and TGF-β1/Smads signaling in diabetic cardiomyopathy. FASEB J..

[168] Kim J.Y., Park J.H., Kim K., Leem J., Park K.K. (2019). Melatonin Inhibits Transforming Growth Factor-β1-Induced Epithelial-Mesenchymal Transition in AML12 Hepatocytes. Biology.

[169] Gutiérrez-Vázquez C., Villarroya-Beltri C., Mittelbrunn M., Sánchez-Madrid F. (2013). Transfer of extracellular vesicles during immune cell-cell interactions. Immunol. Rev..

[170] Buzas E.I. (2023). The roles of extracellular vesicles in the immune system. Nat. Rev. Immunol..

[171] György B., Hung M.E., Breakefield X.O., Leonard J.N. (2015). Therapeutic applications of extracellular vesicles: Clinical promise and open questions. Annu. Rev. Pharmacol. Toxicol..

[172] Buckner J.H., Ziegler S.F. (2004). Regulating the immune system: The induction of regulatory T cells in the periphery. Arthritis Res. Ther..

[173] Nicholson L.B. (2016). The immune system. Essays Biochem..

[174] Poniatowski Ł.A., Wojdasiewicz P., Gasik R., Szukiewicz D. (2015). Transforming growth factor Beta family: Insight into the role of growth factors in regulation of fracture healing biology and potential clinical applications. Mediat. Inflamm..

[175] Liu Z.W., Zhang Y.M., Zhang L.Y., Zhou T., Li Y.Y., Zhou G.C., Miao Z.M., Shang M., He J.P., Ding N. (2022). Duality of Interactions Between TGF-β and TNF-α During Tumor Formation. Front. Immunol..

[176] Chen Z., Zhai J., Ma J., Chen P., Lin W., Zhang W., Xiong J., Zhang C., Wei H. (2023). Melatonin-Primed Mesenchymal Stem Cells-Derived Small Extracellular Vesicles Alleviated Neurogenic Erectile Dysfunction by Reversing Phenotypic Modulation. Adv. Healthc. Mater..

[177] Mathiesen A., Hamilton T., Carter N., Brown M., McPheat W., Dobrian A. (2021). Endothelial Extracellular Vesicles: From Keepers of Health to Messengers of Disease. Int. J. Mol. Sci..

[178] Carrascal L., Nunez-Abades P., Ayala A., Cano M. (2018). Role of Melatonin in the Inflammatory Process and its Therapeutic Potential. Curr. Pharm. Des..

[179] Bantounou M., Plascevic J., Galley H.F. (2022). Melatonin and Related Compounds: Antioxidant and Anti-Inflammatory Actions. Antioxidants.

[180] Tordjman S., Chokron S., Delorme R., Charrier A., Bellissant E., Jaafari N., Fougerou C. (2017). Melatonin: Pharmacology, Functions and Therapeutic Benefits. Curr. Neuropharmacol..

[181] Chuffa L.G.d.A., Seiva F.R.F., Novais A.A., Simão V.A., Martín Giménez V.M., Manucha W., Zuccari D.A.P.d.C., Reiter R.J. (2021). Melatonin-Loaded Nanocarriers: New Horizons for Therapeutic Applications. Molecules.

[182] Kim Y.S., Go G., Yun C.W., Yea J.H., Yoon S., Han S.Y., Lee G., Lee M.Y., Lee S.H. (2021). Topical Administration of Melatonin-Loaded Extracellular Vesicle-Mimetic Nanovesicles Improves 2,4-Dinitrofluorobenzene-Induced Atopic Dermatitis. Biomolecules.

[183] Favero G., Franceschetti L., Bonomini F., Rodella L.F., Rezzani R. (2017). Melatonin as an Anti-Inflammatory Agent Modulating Inflammasome Activation. Int. J. Endocrinol..

[184] Zhang D.X., Vu L.T., Ismail N.N., Le M.T.N., Grimson A. (2021). Landscape of extracellular vesicles in the tumour microenvironment: Interactions with stromal cells and with non-cell components, and impacts on metabolic reprogramming, horizontal transfer of neoplastic traits, and the emergence of therapeutic resistance. Semin. Cancer Biol..

[185] Willms E., Cabañas C., Mäger I., Wood M.J.A., Vader P. (2018). Extracellular Vesicle Heterogeneity: Subpopulations, Isolation Techniques, and Diverse Functions in Cancer Progression. Front. Immunol..

[186] Li Q., Cai S., Li M., Salma K.I., Zhou X., Han F., Chen J., Huyan T. (2021). Tumor-Derived Extracellular Vesicles: Their Role in Immune Cells and Immunotherapy. Int. J. Nanomed..

[187] Théry C. (2011). Exosomes: Secreted vesicles and intercellular communications. F1000 Biol. Rep..

[188] Asare-Werehene M., Nakka K., Reunov A. (2020). The exosome-mediated autocrine and paracrine actions of plasma gelsolin in ovarian cancer chemoresistance. Oncogene.

[189] Raposo G., van Niel G., Stahl P.D. (2021). Extracellular vesicles and homeostasis-An emerging field in bioscience research. FASEB Bioadv..

[190] Doyle L.M., Wang M.Z. (2019). Overview of Extracellular Vesicles, Their Origin, Composition, Purpose, and Methods for Exosome Isolation and Analysis. Cells.

[191] Giannecchini S. (2020). Evidence of the Mechanism by Which Polyomaviruses Exploit the Extracellular Vesicle Delivery System during Infection. Viruses.

[192] Kazemi N.Y., Gendrot B., Berishvili E., Markovic S.N., Cohen M. (2021). The Role and Clinical Interest of Extracellular Vesicles in Pregnancy and Ovarian Cancer. Biomedicines.

[193] Pandi-Perumal S.R., Srinivasan V., Maestroni G.J., Cardinali D.P., Poeggeler B., Hardeland R. (2006). Melatonin: Nature’s most versatile biological signal?. FEBS J..

[194] Kostoglou-Athanassiou I. (2013). Therapeutic applications of melatonin. Ther. Adv. Endocrinol. Metab..

[195] Reiter R.J., Tan D.X., Jou M.J., Korkmaz A., Manchester L.C., Paredes S.D. (2008). Biogenic amines in the reduction of oxidative stress: Melatonin and its metabolites. Neuroendocrinol. Lett..

[196] Liu W., Tang P., Wang J., Ye W., Ge X., Rong Y., Ji C., Wang Z., Bai J., Fan J. (2021). Extracellular vesicles derived from melatonin-preconditioned mesenchymal stem cells containing USP29 repair traumatic spinal cord injury by stabilizing NRF2. J. Pineal Res..

[197] Tang Y., Groom K., Chamley L., Chen Q. (2021). Melatonin, a Potential Therapeutic Agent for Preeclampsia, Reduces the Extrusion of Toxic Extracellular Vesicles from Preeclamptic Placentae. Cells.

[198] Tang D., Cao F., Yan C., Fang K., Ma J., Gao L., Sun B., Wang G. (2022). Extracellular Vesicle/Macrophage Axis: Potential Targets for Inflammatory Disease Intervention. Front. Immunol..

[199] Biemmi V., Milano G., Ciullo A., Cervio E., Burrello J., Dei Cas M., Paroni R., Tallone T., Moccetti T., Pedrazzini G. (2020). Inflammatory extracellular vesicles prompt heart dysfunction *via* TRL4-dependent NF-κB activation. Theranostics.

[200] Zhang Y., He F., Chen Z., Su Q., Yan M., Zhang Q., Tan J., Qian L., Han Y. (2019). Melatonin modulates IL-1*β*-induced extracellular matrix remodeling in human nucleus pulposus cells and attenuates rat intervertebral disc degeneration and inflammation. Aging.

[201] Deus C.M., Tavares H., Beatriz M., Mota S., Lopes C. (2022). Mitochondrial Damage-Associated Molecular Patterns Content in Extracellular Vesicles Promotes Early Inflammation in Neurodegenerative Disorders. Cells.

[202] Reiter R.J., Manchester L.C., Tan D.X. (2010). Neurotoxins: Free radical mechanisms and melatonin protection. Curr. Neuropharmacol..

[203] Ferlazzo N., Andolina G., Cannata A., Costanzo M.G., Rizzo V., Currò M., Ientile R., Caccamo D. (2020). Is Melatonin the Cornucopia of the 21st Century?. Antioxidants.

[204] Talib W.H., Alsayed A.R., Abuawad A., Daoud S., Mahmod A.I. (2021). Melatonin in Cancer Treatment: Current Knowledge and Future Opportunities. Molecules.

[205] Qu P., Luo S., Du Y., Zhang Y., Song X., Yuan X., Lin Z., Li Y., Liu E. (2020). Extracellular vesicles and melatonin benefit embryonic develop by regulating reactive oxygen species and 5-methylcytosine. J. Pineal Res..

[206] Buratta S., Tancini B., Sagini K., Delo F., Chiaradia E., Urbanelli L., Emiliani C. (2020). Lysosomal Exocytosis, Exosome Release and Secretory Autophagy: The Autophagic- and Endo-Lysosomal Systems Go Extracellular. Int. J. Mol. Sci..

[207] Wu L.G., Hamid E., Shin W., Chiang H.C. (2014). Exocytosis and endocytosis: Modes, functions, and coupling mechanisms. Annu. Rev. Physiol..

[208] Gerber S.H., Südhof T.C. (2002). Molecular determinants of regulated exocytosis. Diabetes.

[209] Beurel E., Grieco S.F., Jope R.S. (2015). Glycogen synthase kinase-3 (GSK3): Regulation, actions, and diseases. Pharmacol. Ther..

[210] Kim J.Y., Rhim W.K., Woo J., Cha S.G., Park C.G., Han D.K. (2022). The Upregulation of Regenerative Activity for Extracellular Vesicles with Melatonin Modulation in Chemically Defined Media. Int. J. Mol. Sci..

[211] Trifylli E.-M., Kriebardis A.G., Koustas E., Papadopoulos N., Deutsch M., Aloizos G., Fortis S.P., Papageorgiou E.G., Tsagarakis A., Manolakopoulos S. (2022). The Emerging Role of Extracellular Vesicles and Autophagy Machinery in NASH—Future Horizons in NASH Management. Int. J. Mol. Sci..

[212] Xing H., Tan J., Miao Y., Lv Y., Zhang Q. (2021). Crosstalk between exosomes and autophagy: A review of molecular mechanisms and therapies. J. Cell. Mol. Med..

[213] Baixauli F., López-Otín C., Mittelbrunn M. (2014). Exosomes and autophagy: Coordinated mechanisms for the maintenance of cellular fitness. Front. Immunol..

[214] Colletti M., Ceglie D., Di Giannatale A., Nazio F. (2021). Autophagy and Exosomes Relationship in Cancer: Friends or Foes?. Front. Cell. Dev. Biol..

[215] Salimi L., Akbari A., Jabbari N., Mojarad B., Vahhabi A., Szafert S., Kalashani S.A., Soraya H., Nawaz M., Rezaie J. (2020). Synergies in exosomes and autophagy pathways for cellular homeostasis and metastasis of tumor cells. Cell Biosci..

[216] Guo Y., Gil Z. (2022). The Role of Extracellular Vesicles in Cancer-Nerve Crosstalk of the Peripheral Nervous System. Cells.

[217] Guo H., Sadoul R., Gibbings D. (2018). Autophagy-independent effects of autophagy-related-5 (Atg5) on exosome production and metastasis. Mol. Cell. Oncol..

[218] Sagrillo-Fagundes L., Bienvenue-Pariseault J., Vaillancourt C. (2019). Melatonin: The smart molecule that differentially modulates autophagy in tumor and normal placental cells. PLoS ONE.

[219] Chok K.C., Koh R.Y., Ng M.G., Ng P.Y., Chye S.M. (2021). Melatonin Induces Autophagy *via* Reactive Oxygen Species-Mediated Endoplasmic Reticulum Stress Pathway in Colorectal Cancer Cells. Molecules.

[220] Luo F., Sandhu A.F., Rungratanawanich W., Williams G.E., Akbar M., Zhou S., Song B.-J., Wang X. (2020). Melatonin and Autophagy in Aging-Related Neurodegenerative Diseases. Int. J. Mol. Sci..

[221] Leidal A.M., Debnath J. (2021). Emerging roles for the autophagy machinery in extracellular vesicle biogenesis and secretion. FASEB Bioadv..

[222] Blanc L., Vidal M. (2018). New insights into the function of Rab GTPases in the context of exosomal secretion. Small GTPases.

[223] Ostrowski M., Carmo N.B., Krumeich S., Fanget I., Raposo G., Savina A., Moita C.F., Schauer K., Hume A.N., Freitas R.P. (2010). Rab27a and Rab27b control different steps of the exosome secretion pathway. Nat. Cell Biol..

[224] Tan D.-X., Reiter R.J., Zimmerman S., Hardeland R. (2023). Melatonin: Both a Messenger of Darkness and a Participant in the Cellular Actions of Non-Visible Solar Radiation of Near Infrared Light. Biology.

[225] Novais A.A., Chuffa L.G.A., Zuccari D.A.P.C., Reiter R.J. (2021). Exosomes and Melatonin: Where Their Destinies Intersect. Front. Immunol..

[226] Kalra H., Gangoda L., Fonseka P., Chitti S.V., Liem M., Keerthikumar S., Samuel M., Boukouris S., Al Saffar H., Collins C. (2019). Extracellular vesicles containing oncogenic mutant β-catenin activate Wnt signalling pathway in the recipient cells. J. Extracell. Vesicles.

[227] Pournaghi M., Khodavirdilou R., Saadatlou M.A.E., Nasimi F.S., Yousefi S., Mobarak H. (2021). Effect of melatonin on exosomal dynamics in bovine cumulus cells. Process Biochem..

[228] Alzahrani F.A. (2019). Melatonin improves therapeutic potential of mesenchymal stem cells-derived exosomes against renal ischemia-reperfusion injury in rats. Am. J. Transl. Res..

[229] Heo J.S., Lim J.Y., Yoon D.W., Pyo S., Kim J. (2020). Exosome and Melatonin Additively Attenuates Inflammation by Transferring miR-34a, miR-124, and miR-135b. Biomed. Res. Int..

[230] Su Y., Xu C., Cheng W., Zhao Y., Sui L., Zhao Y. (2023). Pretreated Mesenchymal Stem Cells and Their Secretome: Enhanced Immunotherapeutic Strategies. Int. J. Mol. Sci..

[231] Soekmadji C., Riches J.D., Russell P.J., Ruelcke J.E., McPherson S., Wang C., Hovens C.M., Corcoran N.M. (2016). Modulation of paracrine signaling by CD9 positive small extracellular vesicles mediates cellular growth of androgen deprived prostate cancer. Oncotarget.

[232] Hartmann A., Muth C., Dabrowski O., Krasemann S., Glatzel M. (2017). Exosomes and the Prion Protein: More than One Truth. Front. Neurosci..

[233] Liu L., Labani N., Cecon E., Jockers R. (2019). Melatonin Target Proteins: Too Many or Not Enough?. Front. Endocrinol..

